# Toward Genotype-Informed Dosing of Voriconazole: Head-to-Head Simulations Across CYP2C19 Phenotypes Using Population Pharmacokinetic Models

**DOI:** 10.3390/pharmaceutics17111398

**Published:** 2025-10-28

**Authors:** Yeobin Lee, Nai Lee, Su-jin Rhee, Yun Kim

**Affiliations:** 1 College of Pharmacy, Daegu Catholic University, Gyeongsan 38430, Republic of Korea; dlduqls0530@gmail.com (Y.L.); nailee5029@gmail.com (N.L.); 2 Department of Pharmacy and Institute of Pharmaceutical Research, Wonkwang University College of Pharmacy, Iksan 54538, Republic of Korea

**Keywords:** voriconazole, CYP2C19, population pharmacokinetics, therapeutic drug monitoring, model-informed precision dosing

## Abstract

**Background/Objective:** Voriconazole exhibits nonlinear pharmacokinetics and wide interindividual variability driven by CYP2C19 phenotype and clinical covariates, necessitating early therapeutic drug monitoring (TDM). This study aimed to assess how the choice of population pharmacokinetic (PopPK) models influences genotype-stratified voriconazole exposure under a standardized adult regimen, and to delineate model-specific implications for clinical prescribing. **Methods:** Five CYP2C19-informed PopPK models (Yun, Ling, Wang, Dolton, Friberg) were evaluated under one oral dosing scenario with an identical extensive metabolizers (EM)/intermediate metabolizer (IM)/poor metabolizers (PM) cohort; steady-state exposure metrics were compared across models, with sensitivity checks using model-specific cohorts. **Results:** Yun predicted the highest exposures with the steepest EM–IM–PM gradient, suggesting a need for caution against upper-tail exceedance when genotype effects are pronounced. Ling yielded intermediate exposures with a modest gradient, consistent with adult central tendencies, thus supporting its use for standard adult initial dosing. Wang primarily distinguished between EM and PM, proving useful for lower-bound checks where underexposure risk or limited genotype information is a concern. Friberg (and Dolton) demonstrated lower exposures with limited genotype separation, offering insights when persistent underexposure is suspected. **Conclusions:** These model-specific patterns indicate that PopPK model choice can influence initial dose-band selection and the timing of early TDM in routine adult care. Ling can serve as a baseline for standard adult initiation, whereas Yun is appropriate for safety-first scenarios when upper-tail risk from strong genotype effects is anticipated; Wang assists when IM data are lacking or when lower-bound checks are needed. Generalizability beyond standardized adult dosing (e.g., special populations) remains limited.

## 1. Introduction

Voriconazole demonstrates nonlinear pharmacokinetics, primarily attributed to saturable metabolism and auto-inhibition [1,2]. Its systemic exposure shows wide interindividual variability, largely influenced by cytochrome P450 2C19 (CYP2C19) genotype, hepatic function, inflammation, and drug–drug interactions [3,4,5]. Because of its narrow therapeutic window, early therapeutic drug monitoring (TDM) is recommended to optimize efficacy and minimize toxicity [4,6,7].

Genotype-based dosing influences voriconazole concentrations because CYP2C19 genotype primarily determines metabolic clearance, thereby shifting exposure (AUC, trough). The magnitude of this genotypic effect varies considerably depending on population-specific physiology and clinical context. For instance, children often exhibit higher weight-normalized clearance and greater variability; metabolism can be suppressed in critical illness; and altered clearance, bioavailability, or distribution may occur with hepatic dysfunction, obesity, or Extracorporeal Membrane Oxygenation (ECMO)/transplant. Furthermore, modulation by strong inhibitors/inducers and ethnic differences in allele frequencies also contribute to this variability. Consequently, an identical genotype-stratified dose may result in disparate exposures across different populations [2,3].

Across age groups, CYP2C19 genotype shapes voriconazole exposure in a concordant direction: ultrarapid/rapid metabolizers (UM/RM) tend to have lower concentrations, whereas PM exhibit higher exposure. The magnitude of this effect is age-dependent, reflecting developmental maturation of CYP2C19 and age-related pharmacokinetic differences. These patterns are consistent with developmental maturation of CYP2C19 and age-related differences in absorption and distribution (e.g., absorption rate constant k_a_, absorption lag, bioavailability, intercompartmental clearance *Q*) [8].

Dose adjustment is guided by trough concentrations, with a commonly accepted target range of 2.0–5.5 mg/L (sometimes 1.5–5.5 mg/L). Concentrations ≥ 2 mg/L are generally required for efficacy in invasive fungal infections [5,7,9]. The CYP2C19 phenotype strongly influences voriconazole exposure. For instance, rapid or ultrarapid metabolizers often fall below the therapeutic threshold when given standard doses. Conversely, intermediate and poor metabolizers are more likely to exceed the upper therapeutic range and develop toxicity [3,4,6]. Concentration-dependent adverse events have been reported, with central nervous system symptoms occurring at ~4.0–5.5 mg/L and hepatotoxicity risk rising from ~3.5 to 6 mg/L [7]. Accordingly, TDM within 2–5 days of initiation, followed by stepwise dose adjustment that incorporates genotype, interacting drugs, and hepatic/inflammatory status, is recommended [7,9].

Published voriconazole PopPK models provide useful benchmarks; however, cross-study comparability is limited by heterogeneous design choices (dosing regimens, observation windows), divergent structural/error assumptions (e.g., linear vs. Michaelis–Menten elimination; explicit auto-inhibition vs. none), inconsistent phenotype definitions (EM vs. PM only vs. pooled IM + PM), and non-uniform covariate linkages (hepatic function, inflammation, drug–drug interactions). Most assume first-order absorption and a two-compartment disposition, while elimination has been described as linear, nonlinear Michaelis–Menten, or mixed processes [10,11]. Clearance has been associated with CYP2C19 phenotype, hepatic function, inflammatory markers such as C-reactive protein, and co-medication [5,10,11]. Some models also include the N-oxide metabolite to capture metabolic and absorption variability [5]. Given these structural and covariate differences, systematic comparison of model predictions under standardized clinical scenarios is needed [5,10,11].

In this study, we employed a standardized, quantitative framework using NONMEM to perform a genotype-stratified, head-to-head comparison of representative voriconazole PopPK models. This was conducted under a unified oral dosing scenario and a common observation window, thereby isolating model-intrinsic differences and providing quantitative estimates of phenotype-dependent exposure directly comparable across models under identical conditions. Here, we define scenario-based model selection (SBMS) as identifying—within a predefined clinical context the PopPK model whose predictions are most appropriate for that scenario, and we use this to support TDM by indicating the model best suited to the context and by informing early sampling windows and dose-adjustment decisions.

## 2. Materials and Methods

### 2.1. Literature Search and Model Selection

**Figure 1:**
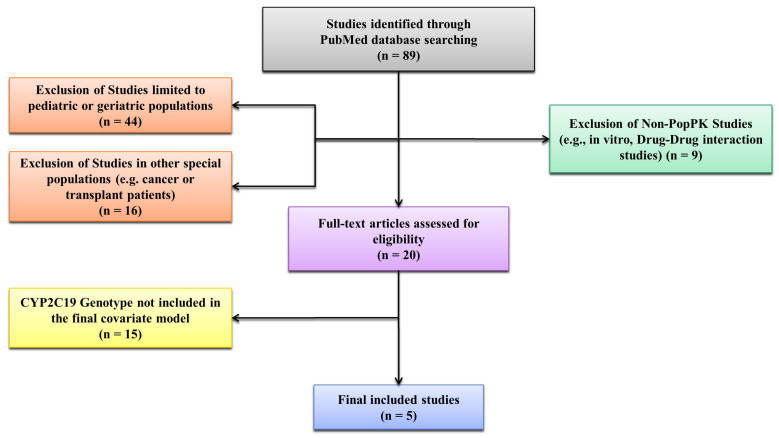
Flowchart of Study Selection for Voriconazole Population Pharmacokinetic Models Incorporating CYP2C19 Genotype.

We systematically searched published PopPK studies of voriconazole. PubMed was searched using combinations of the terms “voriconazole,” “CYP2C19*,*” “CYP3A4*,*” “genotype*,*” “polymorphism*,*” and “population pharmacokinetics” *(*“popPK”*)*. Among 89 records screened, 20 full texts were reviewed, and five studies (Wang, Yun, Ling, Friberg, Dolton) met inclusion criteria (Figure 1). Exclusion criteria were (i) PopPK studies that did not incorporate CYP2C19 genotype or phenotype as a covariate in the final model, (ii) studies limited exclusively to pediatric or elderly cohorts, (iii) studies conducted in highly specialized populations (e.g., transplant recipients, severe organ failure), and (iv) non-PopPK designs [10,11,12,13,14].

### 2.2. Virtual Cohort Generation and Covariates

A virtual cohort of 300 subjects was generated with a balanced CYP2C19 distribution (extensive metabolizers [EM], intermediate metabolizers [IM], poor metabolizers [PM]; 1:1:1 ratio, *n* = 100 each). Demographic and clinical covariates (e.g., age, sex, body weight, liver function) were randomly sampled within the ranges reported in the source publications. When a model did not include a specific covariate, reference values for healthy adults (NIH standards) were imputed. This identical cohort served as the primary dataset and was applied unchanged across all five models to ensure comparability.

### 2.3. Model Implementation and Simulations (Primary and Sensitivity Analyses)

The five PopPK models were implemented according to their published structural and covariate specifications. Simulations were performed in NONMEM (version 7.5.1 Icon Development Solutions, LLC, Gaithersburg, MD, USA) using model-specific subroutines (e.g., ADVAN3 TRANS4 or ADVAN6). For the primary analysis, each model was simulated for a 7-day dosing period consisting of 400 mg oral loading dose twice followed by 200 mg every 12 h (observation window 0–168 h), which approximates near-steady-state conditions for voriconazole in most clinical scenarios. Ten Monte Carlo replicates were conducted for each model, yielding 3000 simulated subjects. Structural details, covariates, and error models are summarized in Table 1. 

Sensitivity analyses were performed using model-specific virtual cohorts (*n* = 40 per model, EM:IM:PM = 1:1:1). Covariates were resampled from the distributions reported in each original study [10,11,12,13,14] (Table 1). The same dosing regimen and simulation settings were applied, with pharmacokinetic profiles and parameters derived from predicted concentrations. 

### 2.4. Exposure Metrics and Genotype Stratification

Noncompartmental analysis (NCA) was performed in R version 4.4.3 using the PKNCA package. Steady-state PK parameters were calculated over a 12 h dosing interval (τ = 12 h) using the linear-up/log-down trapezoidal method. The prespecified PK parameters included:

- AUCτ,ss (area under the plasma concentration–time curve over τ at steady state);
- Ctrough,ss (minimum observed concentration within τ);
- Cavg,ss (average concentration, calculated as AUCτ,ss/τ);
- Cmax,ss (maximum observed concentration within τ);
- CLss/F (apparent clearance at steady state, calculated as Dose/AUCτ,ss); and
- Vss/F (apparent volume of distribution at steady state, derived as CLss/F × MRTss, where MRTss is mean residence time).

CYP2C19 phenotype groups were presented according to the definitions used in the original source models. Specifically, Friberg and Dolton models combined IM and PM into one group versus EM; Wang included only EM and PM; and Yun and Ling reported EM, IM, and PM separately. For consistency, our analyses maintained these groupings. Steady-state exposure metrics were summarized descriptively for each phenotype group as well as for the total simulated population.

### 2.5. Pharmacodynamic Targets and Surrogate

Consistent with current guidance, the pharmacodynamic reference for voriconazole is the free-drug AUC/Minimal inhibitory concentration (MIC) index [15]. In clinical TDM, full AUC workflows are not always feasible; multiple studies show that voriconazole trough concentrations correlate well with AUC and can therefore be used clinically in place of AUC-based targets [16]. Accordingly, we used Ctrough,ss as a pragmatic surrogate and interpreted results in the context of AUC/MIC-anchored targets. The therapeutic trough window was set to 2.0–5.5 mg/L for target attainment, and ≥5.5 mg/L was treated as a toxicity threshold. Under the standardized adult oral regimen, and at near steady state (day 7), For each model and CYP2C19 phenotype (EM/IM/PM), we computed the percentage of simulated trough concentrations that fell within the therapeutic target range (2.0–5.5 mg/L), hereafter referred to as ‘target attainment,’ and the percentage that exceeded this range (≥5.5 mg/L), termed ‘exceedance.

## 3. Results

### 3.1. Simulation Overview

For each model, 3000 virtual subjects were analyzed (10 replicates of a 300-subject primary dataset), with a balanced CYP2C19 distribution (EM:IM:PM = 1:1:1; n = 1000 per phenotype per model).

### 3.2. Concentration–Time Profiles (Primary Analysis)

**Figure 2:**
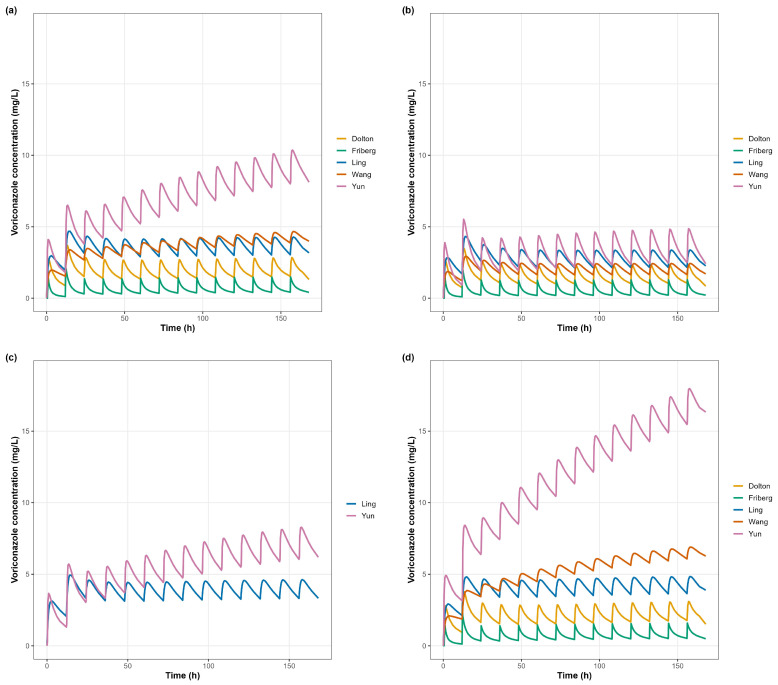
Model-predicted concentration–time profiles of voriconazole by CYP2C19 phenotype (primary dataset). Mean plasma concentration–time profiles simulated using five published PopPK models under identical dosing conditions (400 mg loading, 200 mg q12 h). Panels show (**a**) all subjects, (**b**) extensive metabolizers (EM), (**c**) intermediate metabolizers (IM), (**d**) poor metabolizers (PM). In Friberg and Dolton models, IM and PM were combined as a single group (EM vs. IM + PM). Panels share common axes to allow for side-by-side comparisons of absolute exposure and CYP2C19 phenotype separation across models, ensuring comparability and minimizing scale-related artifacts. Read within each phenotype from left to right; axis limits and time windows are identical across panels.

Under the standard dosing regimen, mean concentration–time profiles showed stable periodicity after the loading phase (Figure 2). Across the 0–168 h window, the overall exposure rank order was Yun > Ling ≈ Wang > Dolton > Friberg, with Yun predicting the highest concentrations and Friberg/Dolton the lowest; Wang and Ling were intermediate.

### 3.3. Primary Exposure Parameters

**Figure 3:**
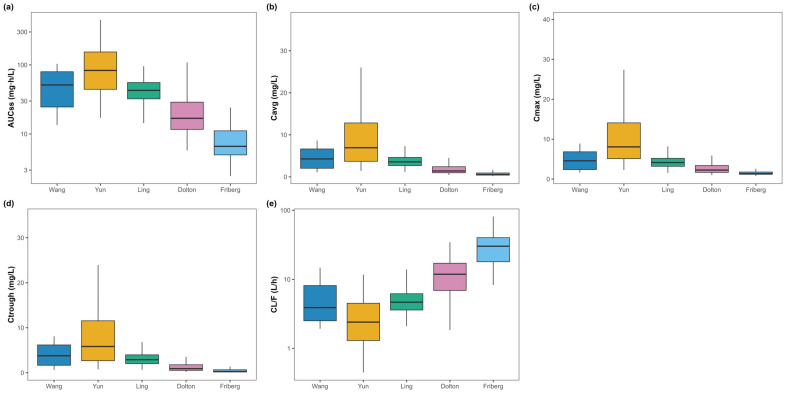
Steady-state pharmacokinetic parameters of voriconazole predicted by five PopPK models (primary dataset). Boxplots of steady-state PK parameters predicted by five PopPK models (Wang, Yun, Ling, Dolton, Friberg) under the standardized dosing regimen. Concentration metrics (mg/L) are shown on a linear scale, whereas exposure/clearance metrics (mg·h/L, L/h) are shown on a logarithmic scale (log10). Sub-figure keys: (**a**) AUCτ,ss (mg·h/L; logarithmic scale, log10), (**b**) Cavg,ss (mg/L; linear scale), (**c**) Cmax,ss (mg/L; linear scale), (**d**) Ctrough,ss (mg/L; linear scale), (**e**) CLss/F (L/h; logarithmic scale, log10).

Steady-state NCA over a 12 h interval (τ = 12 h) confirmed the model-wise rank order (Figure 3, Table 2).

- Yun predicted the highest exposure and lowest clearance (AUCτ,ss 110.47 ± 85.79 mg·h/L; Cavg,ss 9.21 ± 7.15 mg/L; Ctrough,ss 7.96 ± 6.77 mg/L; CLss/F 3.19 ± 2.40 L/h).
- Friberg predicted the lowest exposure and highest clearance (AUCτ,ss 8.34 ± 4.58 mg·h/L; Cavg,ss 0.70 ± 0.38 mg/L; Ctrough,ss 0.42 ± 0.33 mg/L; CLss/F 30.94 ± 14.56 L/h).
- Wang and Ling yielded intermediate exposure (AUCτ,ss 51.91 ± 28.55 and 44.61 ± 15.75 mg·h/L; CLss/F 5.50 ± 3.20 and 5.12 ± 1.98 L/h, respectively).
- Dolton produced lower exposure with mid-to-higher clearance (AUCτ,ss 24.61 ± 20.63 mg·h/L; CLss/F 12.50 ± 6.89 L/h).

Inter-model exposure differed by an order of magnitude (e.g., Yun vs. Friberg), indicating that the choice of PopPK model materially changes simulated target attainment and risk classification under the same regimen.

### 3.4. Genotype-Stratified Exposure (Primary Analysis)

**Figure 4:**
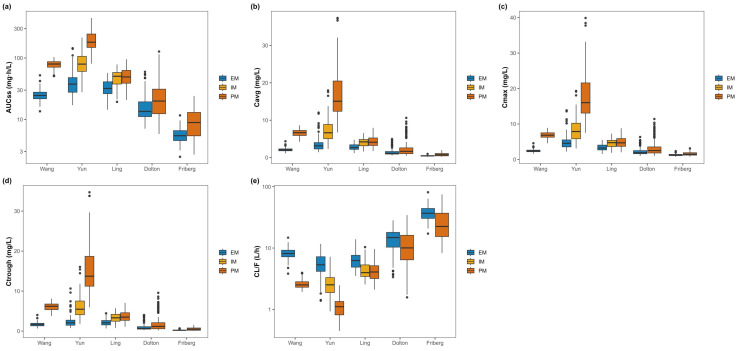
Model-predicted steady-state pharmacokinetic parameters of voriconazole by CYP2C19 phenotype (primary dataset). (a) AUCτ,ss (mg·h/L; logarithmic scale, log10), (b) Cmax,ss (mg/L; linear scale), (c) Cavg,ss (mg/L; linear scale), (d) Ctrough,ss (mg/L; linear scale), (e) CLss/F (L/h; logarithmic scale, log10).Panels share common axes to allow for side-by-side comparisons of absolute exposure and CYP2C19 phenotype separation across models, ensuring comparability and minimizing scale-related artifacts. Read within each phenotype from left to right; axis limits and time windows are identical across panels.

Genotype strata followed the original model definitions (Friberg/Dolton: IM + PM vs. EM; Wang: EM and PM; Yun/Ling: EM, IM, PM). Descriptive steady-state metrics are summarized in Figure 4 and Table 2.

- Yun showed a clear stepwise increase in exposure from EM → IM → PM with a reciprocal decline in CLss/F (AUCτ,ss: 42.64 ± 23.12, 86.52 ± 37.24, 202.25 ± 81.08 mg·h/L; Ctrough,ss: 2.49 ± 1.65, 6.05 ± 2.79, 15.34 ± 6.25 mg/L; CLss/F: 5.69 ± 2.27, 2.27 ± 1.15, 1.15 ± 0.45 L/h for EM, IM, PM).
- Ling exhibited the same directionality with a modest gradient (AUCτ,ss EM 33.70 ± 9.92, IM 48.35 ± 13.80, PM 51.78 ± 16.56 mg·h/L; CLss/F EM 6.49 ± 2.06, IM 4.56 ± 1.59, PM 4.29 ± 1.47 L/h).
- Wang: PM exceeded EM in exposure with lower clearance (AUCτ,ss 24.90 ± 5.73 vs. 78.92 ± 11.49 mg·h/L; Ctrough,ss 1.65 ± 0.53 vs. 6.05 ± 0.98 mg/L; CLss/F 8.42 ± 1.81 vs. 2.59 ± 0.41 L/h for EM vs. PM).
- Friberg and Dolton: separation between (IM + PM) and EM was limited but directionally consistent (e.g., Dolton AUCτ,ss 27.70 ± 23.12 vs. 18.42 ± 12.40 mg·h/L; Friberg 9.72 ± 4.95 vs. 5.59 ± 1.66 mg·h/L for IM + PM vs. EM).

Relative to the therapeutic range (2.0–5.5 mg/L), the primary dataset suggests a high probability of supratherapeutic Ctrough,ss in Yun IM/PM, subtherapeutic Ctrough,ss in Wang EM and across most Friberg strata, and improved attainment with increasing metabolizer impairment (EM → IM → PM) in Ling. These patterns the need for early TDM and genotype-aware dosing under standard dosing.

### 3.5. Secondary Exposure Parameters

Trends in Cmax,ss, CLss/F, and Vss/F paralleled the primary parameters (Figure 3, Figure 4, Table 2). The lowest and highest CLss/F were observed in Yun PM and Friberg EM, respectively.

### 3.6. Therapeutic Target Attainment

**Figure 5:**
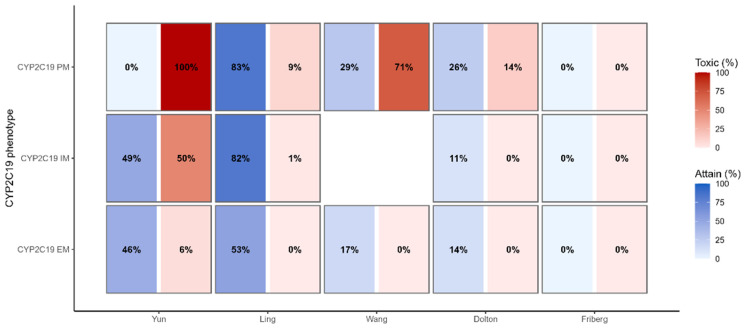
Therapeutic Target Attainment and Toxicity by Model and CYP2C19 Phenotype. left (blue) shows the percentage of simulated troughs within the therapeutic window (2.0–5.5 mg/L), and right (red) shows the percentage ≥ 5.5 mg/L (toxicity). Rows are CYP2C19 phenotypes (EM/IM/PM) and columns are published PopPK models (Yun, Ling, Wang, Dolton, Friberg). Percentages are read at near steady state (day 7) under the standardized adult oral regimen.

Using Ctrough,ss-based Therapeutic targets under the standardized adult regimen, model choice altered the balance between therapeutic-window occupancy and toxicity across CYP2C19 phenotypes (Figure 5). Ling maintained high within-window occupancy with minimal toxicity across EM/IM/PM, supporting a standard adult start with routine early TDM. Yun shifted IM and PM toward toxicity under the same regimen, indicating caution and favoring a conservative initial dose with very early verification. Wang primarily improved lower-bound coverage when underexposure is likely or genotype information is limited, justifying a more assertive start with close early sampling; in PM, however, its tendency toward higher toxicity warrants prompt dose reduction if elevated troughs are observed. Dolton was conservative (low within-window occupancy, low toxicity), and Friberg minimized toxicity at the expense of within-window occupancy, aligning with scenarios in which upper-bound control is prioritized.

**Figure 6:**
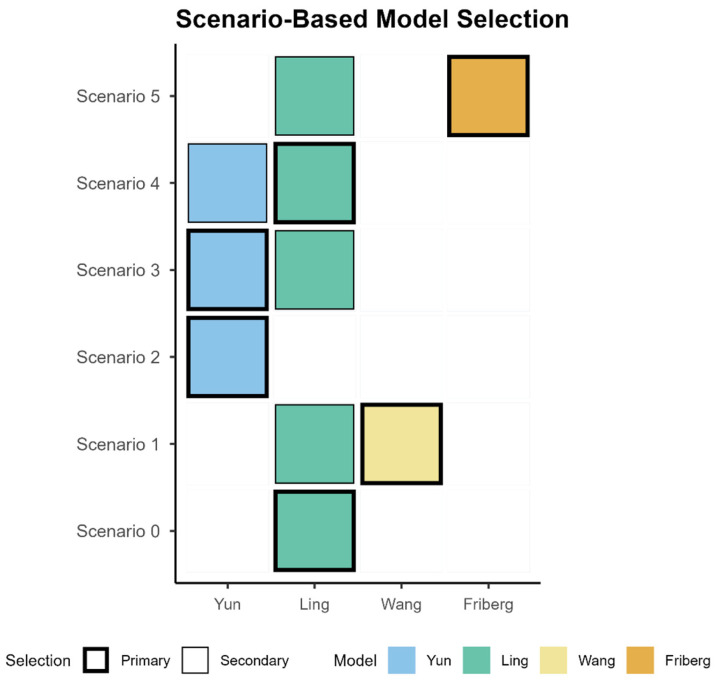
Scenario-Based Model Selection (SBMS) Matrix. Colored cells indicate the model (s) recommended for that scenario; a bold outline marks the Primary model, and any additional filled cell in the same row denotes a Secondary model used for supportive checks or adjustments.

Taken together, these target attainment patterns underpin the SBMS (Figure 6) and are translated into concrete prescribing actions starting dose band, timing of early TDM, and the direction/magnitude of the first adjustment (Table 3).

To operationalize model choice by clinical context, we present SBMS in which one PopPK model is recommended per predefined scenario (Figure 6), and Table 3 links each scenario to prescribing/TDM steps (starting-dose band, timing of early TDM, and the direction/magnitude of the first adjustment). In a standard adult start, Ling is recommended because it maintains therapeutic-window exposure with minimal toxicity. When underexposure is likely or genotype information is limited, Wang is preferred to secure the lower bound and is paired with early trough verification. When upper-bound control is prioritized (e.g., hepatotoxicity concern or CYP2C19 IM/PM), Yun is recommended with a conservative start and very early TDM. When covariate burden (e.g., inflammation, low albumin, advanced age) suggests reduced clearance and increased exposure, Ling is used with dose reduction or interval extension and early confirmation. When persistent underexposure is suspected, Friberg is selected to raise exposure, followed by close TDM. Together, the matrix specifies which model to use in each scenario and how that choice is implemented at the bedside.

### 3.7. Sensitivity Analyses (Model-Specific Cohorts)

Sensitivity analyses using model-specific virtual cohorts (covariates resampled from each source study) reproduced the primary rank order (Yun > Ling ≈ Wang > Dolton > Friberg) across 0–168 h. CYP2C19 phenotype patterns were preserved: in Yun, exposure increased EM → IM → PM with a reciprocal decline in CLss/F; Ling showed the same directionality with a modest gradient; Wang showed higher exposure/lower CLss/F in PM than EM. In Friberg and Dolton, separation between (IM + PM) and EM remained limited but directionally consistent. Compared with the primary analysis, distributions widened (reflecting broader covariate dispersion), while central tendencies remained similar. Numerical summaries are provided in Table S1, and distributions are shown in Figures S1–S3.

## 4. Discussion

Under a standardized oral regimen (400 mg loading followed by 200 mg q12 h), the five published PopPK models yielded materially different near–steady-state exposures and distinct CYP2C19 phenotype gradients. The primary analysis ranked overall exposure as Yun > Ling ≈ Wang > Dolton > Friberg, consistent with voriconazole’s nonlinear elimination (saturable metabolism, auto-inhibition) and narrow therapeutic window. These head-to-head simulations provide a unified quantitative frame for interpreting genotype-stratified exposure under one dosing design, complementing guidance that advocates early TDM (typically within 2–5 days) and trough-guided dose adjustment in clinical practice [7,17,18].

Adult voriconazole PopPK commonly reports typical clearances of ~3–5 L/h in general populations, with phenotype-linked decreases from EM to IM to PM. In our simulations, Ling reproduced a modest yet consistent EM→IM→PM gradient and maintained trough central tendencies closer to the commonly cited therapeutic window (≈2.0–5.5 mg/L), aligning with literature-based central tendencies and meta-analytic gradients (higher troughs in IM/PM vs. EM). Yun predicted substantially higher exposure and a steeper phenotype gradient (CLss/F EM→PM: ~5.7→1.2 L/h), a profile that may anticipate upper-range exceedance in PM under standard dosing. Wang (EM vs. PM only) accentuated the EM–PM separation but lacked an IM stratum, limiting coverage across the full phenotype spectrum. Dolton and Friberg predicted lower absolute exposures with limited separation between EM and IM + PM, which may under-represent genotype effects when these are clinically prominent. Collectively, these differences indicate that “model choice” can shift simulated target attainment and risk classification under identical dosing [5,6,14,19,20].

The observed model divergence plausibly stems from variations in (i) elimination specifications (e.g., linear, Michaelis–Menten, or mixed kinetics; explicit auto-inhibition), (ii) absorption/distribution choices and residual-error structures, (iii) the magnitude and shrinkage of inter-individual variability, and (iv) covariate parameterization. This includes, notably, how CYP2C19 is linked and whether factors such as C-reactive protein (inflammation), hepatic function, and drug–drug interactions modulate CL and/or F [18]. Because our comparisons use apparent clearance (CLss/F), between-model differences may arise from assumptions on both CL and bioavailability (F). Thus, absolute scale disagreements do not necessarily imply inferior model quality; rather, they may reflect population context, covariate distributions, and identifiability trade-offs in the original developments. This perspective argues for “fit-for-purpose” model selection rather than a single model as universally superior.

Ling, which tracks adult central tendencies and encodes an inflammation effect, appears suitable as a baseline model for initial dosing and TDM interpretation in general adult settings. Yun, with its steeper genotype effect and higher predicted exposures, may be safety-first in scenarios where pronounced phenotype effects or hepatic dysfunction are anticipated—provided that initial dose reduction and early TDM are applied. Wang can bound EM–PM contrasts when IM status is unavailable, though less suited to estimating central tendencies. Dolton and Friberg may offer conservative starting estimates where genotype effects are muted or uncertain, but reliance on TDM increases if genotype-driven exposure is expected to be clinically decisive. Importantly, our simulations highlight that early TDM and genotype-aware titration remain central to mitigating both subtherapeutic exposure and toxicity risk under standard dosing [5,6,11,18,19,20,21,22].

Sensitivity analyses using model-specific virtual cohorts (covariates resampled from each source study) reproduced the primary rank order and phenotype patterns while widening distributions—a predictable effect of broader covariate dispersion. Central tendencies remained similar, supporting that the qualitative conclusions are not driven solely by the unified primary dataset. This robustness across scenario sets strengthens confidence in the comparative patterns, while also illustrating how population context can broaden clinical prediction intervals around a given model’s exposure.

There are several limitations and future directions in this study. First, although dosing, cohort, and readout windows were standardized, we deliberately retained each model’s original structural and error specifications (e.g., nonlinearity/auto-inhibition, absorption lag, variability) rather than re-estimating under a shared structure; this may have contributed to the observed rank order and the apparent magnitude of phenotype separation. Second, parameter uncertainty and η-shrinkage from the source models were not propagated through full simulation–estimation cycles, which could narrow predictive spread and affect calibration. Third, phenotype groupings followed source models (e.g., IM + PM vs. EM; EM vs. PM), which limits direct cross-model ratio metrics but reflects how these models are actually specified and used. Finally, the SBMS is offered as an interpretive aid; the prescribing suggestions it informs warrant prospective, TDM-anchored verification. We also have de-identified voriconazole TDM datasets and will use them for a follow-up external validation, assessing bias/precision calibration, and target attainment across relevant clinical settings.

## 5. Conclusions

Under a standardized oral regimen, five published voriconazole PopPK models yielded distinct near-steady-state exposures and CYP2C19 phenotype gradients. This indicates that the choice of model substantially influences simulated target attainment and risk classification. Ling most closely tracked adult central tendencies with a modest, literature-consistent EM→IM→PM gradient (a practical baseline for initial dosing and TDM interpretation), whereas Yun predicted higher exposures with a steeper genotype effect (a safety-first option when upper-range risk is anticipated, paired with early TDM and dose reduction). Wang informs EM–PM contrasts but lacks an IM stratum, and Dolton/Friberg showed modest phenotype separation that may understate genotype dependence, reinforcing reliance on TDM. Sensitivity analyses using model-specific virtual cohorts preserved the primary rank order and phenotype patterns while widening distributions, supporting the robustness of qualitative conclusions across population contexts. Next, our group will conduct external clinical validation using our real-world, genotype-linked TDM dataset—stratified by inflammation and hepatic function—and extend to PK/PD target-attainment and Bayesian forecasting to refine genotype-guided dosing. However, generalizability is limited to adult standardized dosing. Therefore, prospective external validation using real TDM datasets, which involves assessing bias/precision, calibration, and target attainment, is strongly warranted.

## Figures and Tables

**Table 1:** Voriconazole population pharmacokinetic models included in the analysis: structural model, elimination mechanism, covariates (including CYP2C19 linkage), residual error model, and source population characteristics.

Study	N (Male/Female)	Subject Characteristics (n)	Age	Body Weight	CYP2C19 Phenotype	Samples		Data	Structural Model	Pharmacokinetic Parameters	Model Variability	Covariates
						Per Subject	Total					
Friberg et al. [11]	173 (101/72)	Immunocompromised children (112), adolescents (26), and healthy adults (35)	12.9 (2–55)	38.7 (10.8–97)	EM (98) IM (66) UM (4) PM (5)	19.3	3336	Rich data from five PK studies	2-Compartment model with first-order oral absorption, with a lag time, and mixed linear and Michaelis–Menten elimination	Ka = 100 h^−1^ (fixed) [adults] Ka = 1.19 h^−1^ (children) Ka = 1.19 × (1 − 0.615 × ADO) h^−1^ (pediatrics) F = 0.642 Lag time = 0.949 h (adults) Lag time = 0.12 h (pediatrics) V1 = 79.0 × (WT/70) L V2 = 103 × (WT/70) L Q = 15.5 × (WT/70)^0.75^ L/h (adults) Q = 15.5 × (WT/70)^0.75^ × (1 + 0.637) L/h (pediatrics) CL = 6.16 × (WT/70)^0.75^ L/h Vmax,1 = 114 × (WT/70)^0.75^ mg/h Vmax,inh = 0.82 (adults/adolescents) Vmax,inh = 0.75 (children) T50 = 2.41 h Vmax = Vmax,1 × {1 − Vmax,inh × (T − 1)/[(T − 1) + (T50 − 1)]} mg/h Km = 1.15 mg/L	BSV Ka = 89.8% (pediatrics) BSV logit (F) = 0.78 (adults) BSV logit (F) = 2.3 (pediatrics) BSV V1 = 14% BSV V2 = 77% BSV Q = 42.4% BSV CL = 44% (adults) BSV CL = 75% (pediatrics) BSV Vmax,1 = 79% (adults) BSV Vmax,1 = 24% (children) BSV Vmax,1 = 28% (adolescents) BSV Km = 136% Proportional REE = 37% to 59%	V1: WT V2: WT Q: WT CL: WT Vmax,1: WT Vmax,inh: Study population (children or adolescents) Ka: Study population (adolescents only, ADO = 1) Lag time: Study population (adults vs. pediatrics)
Wang et al. [12]	151 (104/47)	Invasive fungal infection patients (n = 151)	59 (18–99)	59.1 (35.0–80.0)	EM (64) IM (65) PM (19) UM (3)	4	406	Sparse sampling	1-Compartment model with first-order absorption and elimination	Ka = 1.1 h^−1^ (fixed) F = 0.895 V = 200 × [1 − 0.0098 × (AGE − 61)] L CL = 6.95 × [1 − 0.012 × (AGE − 61)] × (1 − 0.37 × PM) × [1 − 0.0016 × (ALP − 104)] L/h	BSV F = 18.9% BSV V = 25.4% BSV CL = 28.7% Proportional REE = 10.8% Additive REE = 0.016 mg/L	V: AGE CL: AGE, CYP2C19 genotype (PM), ALP
Dolton et al. [10]	240 (152/88)	Healthy adults (63) and adult patients with fungal infection or at risk for fungal infections (177)	34 (18–88)	69 (39–115)	EM&RM (56) IM&PM (38) UM (146)	14	3352	Rich data from five PK studies and sparse data from a TDM study	2-Compartment model with first-order oral absorption and Michaelis–Menten elimination	Ka = 0.53 h^−1^ Lag_time = 0.162 h F = 0.942 L V1 = 27.1 L V2 = 127 L Q = 35.1 L/h Km = 3.33 mg/L Vmax = 43.9 × (1 − 0.412 × CYP2C19) × (1 − 0.429 × RIT) × (1 + 1.07 × SJW) × (1 + 2.03 × POR) × (1 + 0.366 × POP) × (1 + 0.564 × MET) × (1 + 0.557 × DEX) × (1 + 1.11 × HV)	BSV Ka = 41.6% BSV F = 36.7% BSV V1 = 83.4% BSV V2 = 38.1% BSV Vmax = 26.8% BSV Km = 64.5% Prop REE = 33.8% Add REE = 0.005 mg/L	Vmax: CYP2C19 genotype, short-term ritonavir, St John’s wort, phenytoin, rifampicin, glucocorticoids (prednisone, prednisolone, methylprednisolone, dexamethasone), study population (healthy volunteers)
Yun et al. [13]	193 (164/29)	Healthy Subjects (93) Patients (100)	34 (18–80)	66.0 (40.8–88.5)	EM (75), IM (70), PM (48)	9.5	1828	Rich data from a study	3-compartment model with a first-order oral absorption, lag time, and elimination along with an inhibition compartment model	Ka = 1.23 h^−1^ F1 = 0.876 ALAG1 = 0.237 h CL = 45.3 L/h V2 = 35.7 L V3 = 58.9 L Q2 = 10.9 L/h V4 = 25.4 L Q3 = 54.6 L/h RCLF = 0.162 KIC = 0.002 µM^−1^ CL0 = 45.3 × (WT/70)^0.595 × (1 − 0.186·IM − 0.746·PM) × (1 − 0.75·LIVER) INH = RCLF + (1 − RCLF) × (1 − (Cinh/(IC50 + Cinh))) CL = CL0 × INH	BSV CL = 21.4% BSV V2 = 40.2% BSV V3 = 20.6% BSV Q2 = 28.8% BSV KA = 87.8% BSV F1 = 84.4% BSV RCLF = 54.4% Add REE (healthy) 0.208 mg/L Add REE (patient) 0.799 mg/L	CL: CYP2C19 phenotype, WT, liver dysfunction (grade 3) RCLF:CYP2C19 phenotype V3: WT Q2: WT
Ling et al. [14]	167 (119/48)	Patients with invasive fungal infections	68.87 ± 14.87 (71.16–97)	64.48 ± 12.24 (65.37–100)	EM (66), IM (72) PM (29)	1.4	232	Rich data from a study	1-Compartment model with first-order absorption and elimination	Ka = 1.10 h^−1^ (fixed) F = 0.965 V = 134 × (WT/65)^(2.21)^ L CL = 3.83 L/h × 0.794^(IM)^ × 0.635^(PM)^ × CRP^(−0.153)^; apply to EM/IM only × (ALB/34.8)^(0.664)^ × (AGE/71)^(−0.582)^ × 1.41^(Female)^	BSV CL = 38.9% BSV V = 45.2% Prop REE = 14.7% Add REE = 0.58 µg/mL	CL:CYP2C19 phenotype (IM, PM) ALB AGE Gender WT V: WT

RM, Rapid metabolizer; EM, Extensive metabolizer; IM, Intermediate metabolizer; PM, Poor metabolizer; UM, Unknown metabolizer; BSV, Between subject variability; REE, Residual error; Ka, first-order absorption rate constant; F, oral bioavailability; V: volume of distribution; Q, intercompartmental clearance between central and peripheral compartments; WT, body weight; CL, clearance; Vmax, maximal metabolic rate (Michaelis–Menten); Vmax,inh, maximal rate/capacity of auto-inhibition (time-dependent inhibition component); Km, Michaelis–Menten constant (substrate concentration at half Vmax); ALP, alkaline phosphatase; RCLF, remaining CL fraction reflecting the CL at a steady state and the fraction that cannot be inhibited; KIC, inhibition constant for auto-inhibition (concentration for half-maximal inhibition); Cinh, concentration in the inhibition compartment; IC50, Cinh yielding 50% of maximum CL inhibition; CRP, C-reactive protein; ALB, albumin.

**Table 2:** Steady-state pharmacokinetics of voriconazole across population PK models based on a primary dataset using individual predicted concentrations.

Model	CYP2C19 Phenotype	AUCτ,ss (mg·h/L)	Cavg,ss (mg/L)	Cmax,ss (mg/L)	Ctrough,ss (mg/L)	CLss/F (L/h)	Vss/F (L)
Friberg	Total	8.34 ± 4.58	0.70 ± 0.38	1.48 ± 0.44	0.42 ± 0.33	30.94 ± 14.56	134.43 ± 50.62
	EM	5.59 ± 1.66	0.47 ± 0.14	1.28 ± 0.29	0.22 ± 0.11	39.05 ± 11.88	162.85 ± 40.97
	IM + PM	9.72 ± 4.95	0.81 ± 0.41	1.58 ± 0.47	0.52 ± 0.36	26.89 ± 14.10	120.22 ± 49.05
Wang	Total	51.91 ± 28.55	4.33 ± 2.38	4.66 ± 2.34	3.85 ± 2.34	5.50 ± 3.20	31.24 ± 17.22
	EM	24.90 ± 5.73	2.07 ± 0.48	2.43 ± 0.42	1.65 ± 0.53	8.42 ± 1.81	47.18 ± 8.81
	PM	78.92 ± 11.49	6.58 ± 0.96	6.88 ± 0.90	6.05 ± 0.98	2.59 ± 0.41	15.30 ± 2.29
Dolton	Total	24.61 ± 20.63	2.05 ± 1.72	2.85 ± 1.76	1.59 ± 1.59	12.50 ± 6.89	62.05 ± 30.48
	EM	18.42 ± 12.40	1.53 ± 1.03	2.34 ± 1.10	1.04 ± 0.94	14.48 ± 6.31	70.94 ± 27.27
	IM + PM	27.70 ± 23.12	2.31 ± 1.93	3.10 ± 1.97	1.76 ± 1.79	11.51 ± 6.97	57.60 ± 30.89
Yun	Total	110.47 ± 85.79	9.21 ± 7.15	10.39 ± 7.19	7.96 ± 6.77	3.19 ± 2.40	17.44 ± 12.22
	EM	42.64 ± 23.12	3.55 ± 1.93	4.93 ± 2.18	2.49 ± 1.65	5.69 ± 2.27	29.99 ± 11.33
	IM	86.52 ± 37.24	7.21 ± 3.10	8.34 ± 3.30	6.05 ± 2.79	2.27 ± 1.15	15.57 ± 6.41
	PM	202.25 ± 81.08	16.85 ± 6.76	17.90 ± 7.01	15.34 ± 6.25	1.15 ± 0.45	6.77 ± 2.63
Ling	Total	44.61 ± 15.75	3.72 ± 1.31	4.28 ± 1.37	3.08 ± 1.27	5.12 ± 1.98	28.80 ± 10.55
	EM	33.70 ± 9.92	2.81 ± 0.83	3.37 ± 0.89	2.16 ± 0.80	6.49 ± 2.06	36.07 ± 10.81
	IM	48.35 ± 13.80	4.03 ± 1.15	4.63 ± 1.21	3.31 ± 1.15	4.56 ± 1.59	25.74 ± 8.49
	PM	51.78 ± 16.56	4.31 ± 1.38	4.84 ± 1.46	3.65 ± 1.29	4.29 ± 1.47	24.59 ± 8.15

Values are mean ± standard deviation from standard oral dosing regimen (400 mg twice followed by 200 mg twice daily). AUCτ,ss, area under the concentration–time curve at steady state; Cavg,ss, average concentration (AUCss/τ); Cmax, maximum concentration; Ctrough,ss, trough concentration; CLss/F, apparent clearance; Vss/F, apparent steady-state volume of distribution.

**Table 3:** Scenario-to-Model Mapping and Prescribing with TDM Actions.

Scenario	Description	Model	Prescribing
Scenario 0	Adult standard start, safety-first	Primary: Ling	Start with standard dosing and early TDM
Scenario 1	Limited genotype info (EM vs. PM only), need lower/upper-bound check	Primary: Wang Secondary: Ling	Define phenotype-specific assumptions for the therapeutic window and verify with early TDM; adjust if attainment or exceedance is observed.
Scenario 2	Hepatotoxicity concern or rising liver enzymes (e.g., ALT/AST) under the standardized regimen; prioritization of upper-bound control.	Primary: Yun	Reduce dose or extend dosing interval to control the upper bound; intensify TDM to confirm trough ≤5.5 mg/L; Continue close LFT monitoring and re-titrate if needed.
Scenario 3	Genotype confirmed	Primary: Yun Secondary: Ling	Conservative start or interval extension for IM/PM, uptitrate EM if below target—TDM-guided
Scenario 4	Inflammation or covariate-anchored adjustment (CRP increased, low ALB, elderly)	Primary: Ling Secondary: Yun	Reduce dose or extend interval per covariates
Scenario 5	Suspected underexposure	Primary: Friberg Secondary: Ling	If below target, uptitrate by 20–30%

Each row defines a predefined clinical scenario and the recommended PopPK model (s) (Primary, and when applicable Secondary), with the corresponding prescribing/TDM steps: starting-dose band (conservative vs. standard), timing of early TDM, and the direction/magnitude of the first dose or interval adjustment.

## Data Availability

Data supporting the findings of this study are available from the corresponding author upon request.

## Supplementary Materials

The following supporting information can be downloaded at: https://www.mdpi.com/article/10.3390/pharmaceutics17111398/s1, Figure S1: Model-predicted voriconazole concentration–time profiles stratified by CYP2C19 genotype based on a sensitivity dataset.; Figure S2: Distribution of steady-state pharmacokinetic parameters predicted from individual predictions across voriconazole population pharmacokinetic models using a sensitivity dataset.; Figure S3: Model-based distributions of steady-state pharmacokinetic parameters by CYP2C19 phenotype based on individual predictions using a sensitivity dataset.; Table S1: Non-compartmental analysis of steady-state pharmacokinetics of voriconazole across population PK models based on a sensitivity dataset using individual predicted concentrations.
