# Supplementary material for: Toward Genotype-Informed Dosing of Voriconazole: Head-to-Head Simulations Across CYP2C19 Phenotypes Using Population Pharmacokinetic Models
Source: Pharmaceutics. 2025 Oct 28;17(11):1398. doi: 10.3390/pharmaceutics17111398 (PMC12655191; doi:10.3390/pharmaceutics17111398)
Supplement: Supplementary file 1 [file pharmaceutics-17-01398-s001.zip › pharmaceutics-3925023-supplementary.pdf]

**Supplementary Figure S1. Model-predicted voriconazole concentration–time profiles stratified by CYP2C19 genotype based on a sensitivity dataset.**

Each panel displays the mean predicted plasma concentration–time profile of voriconazole simulated using five published population pharmacokinetic models under identical standard dosing conditions. Panels represent: (a) all subjects, (b) extensive metabolizers (EM), (c) intermediate metabolizers (IM), (d) poor metabolizers (PM). Note: In Friberg and Dolton models, IM and PM were combined as one group (NM vs IM+PM).

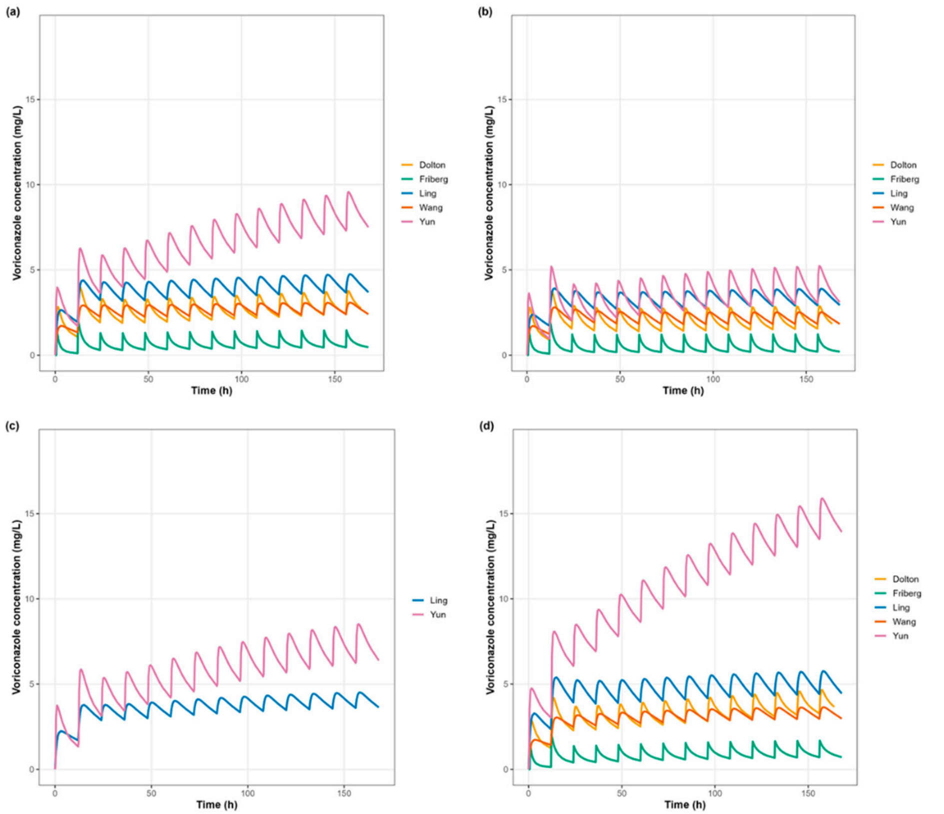

**Supplementary Figure S2. Distribution of steady-state pharmacokinetic parameters predicted from individual predictions across voriconazole population pharmacokinetic models using a sensitivity dataset.**

Boxplots of steady-state pharmacokinetic parameters of voriconazole predicted by five population pharmacokinetic models (Wang, Yun, Ling, Dolton, Friberg). Concentration-based parameters (Ctrough, Cavg, Cmax; mg/L) are presented on a linear scale, while exposure (AUCss; mg·h/L) and apparent clearance (CL/F; L/h) are displayed on a logarithmic scale to accommodate the wider inter-model and inter-genotype variability.

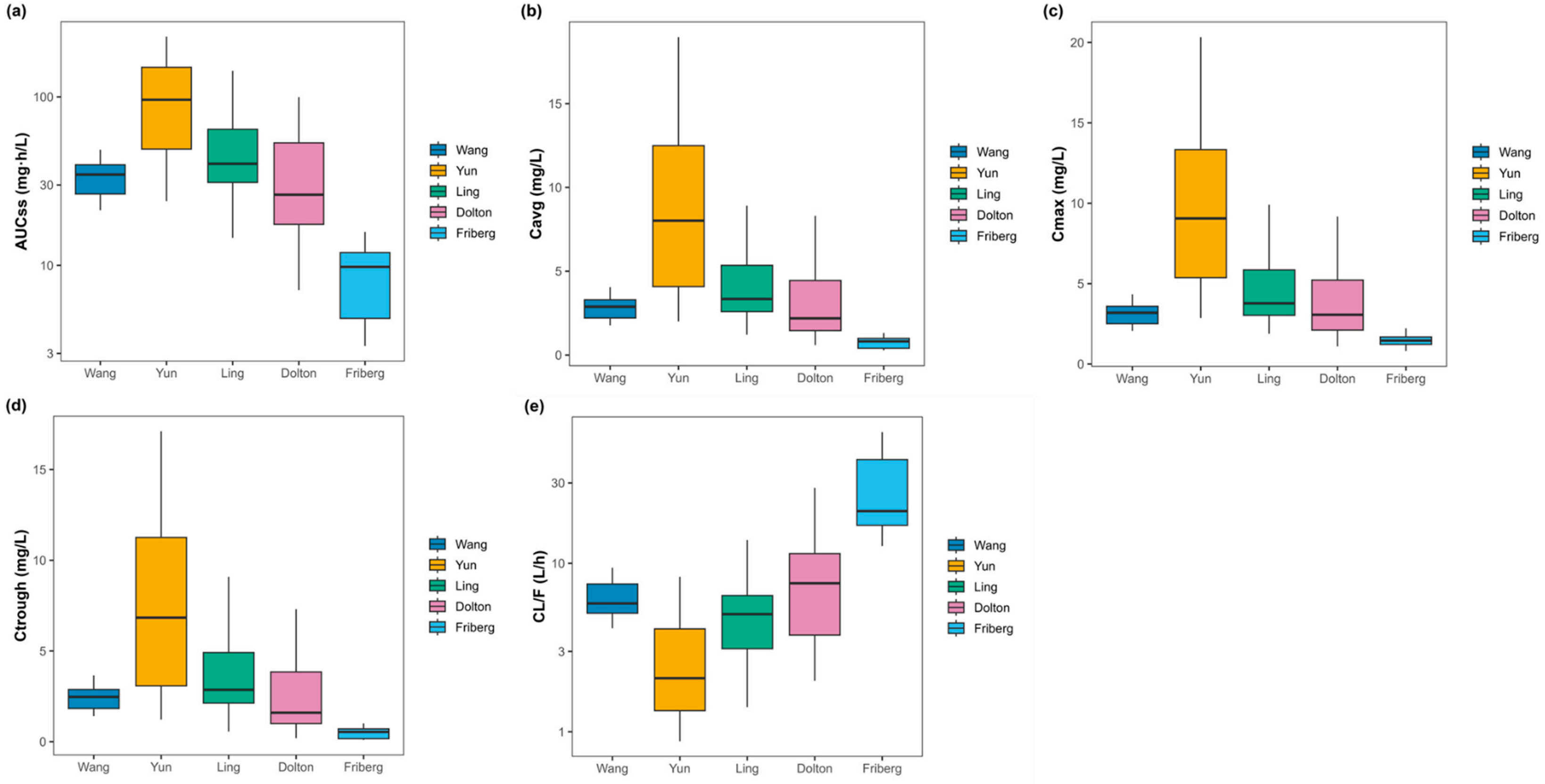

**Supplementary Figure S3. Model-based distributions of steady-state pharmacokinetic parameters by CYP2C19 phenotype based on individual predictions using a sensitivity dataset.**

Boxplots of steady-state pharmacokinetic parameters of voriconazole predicted by five population pharmacokinetic models (Wang, Yun, Ling, Dolton, Friberg). Concentration-based parameters (Ctrough, Cavg, Cmax; mg/L) are presented on a linear scale, while exposure (AUCss; mg·h/L) and apparent clearance (CL/F; L/h) are displayed on a logarithmic scale to accommodate the wider inter-model and inter-genotype variability.

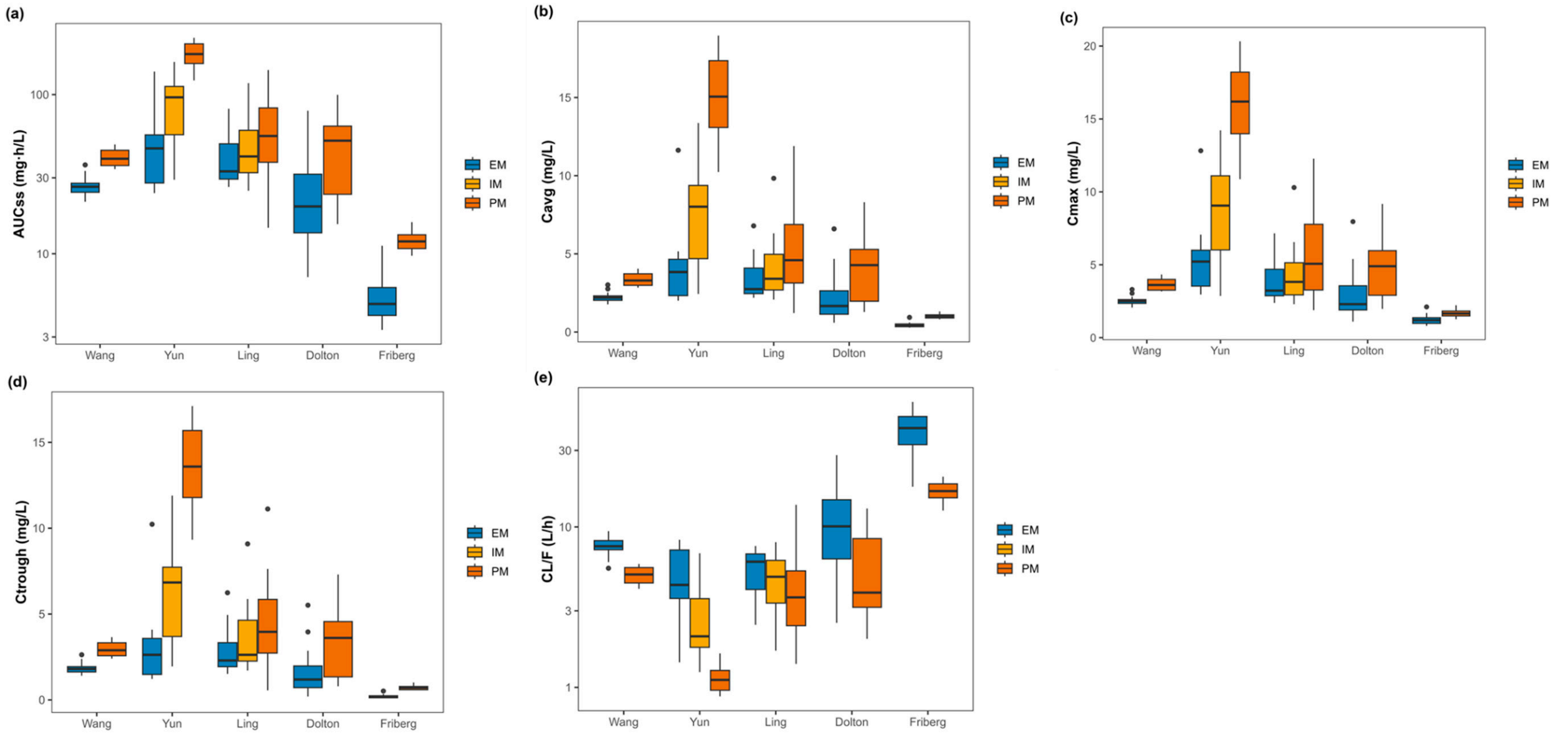

**Supplementary Table S1. Non-compartmental analysis of steady-state pharmacokinetics of voriconazole across population PK models based on a sensitivity dataset using individual predicted concentrations.**

| Model | GENO | AUCss (mg·h/L) | Cavg (mg/L) | Cmax (mg/L) | Ctrough (mg/L) | CL/F (L/h) | Vss/F (L) |
|---|---|---|---|---|---|---|---|
| Friberg | Total | 8.74 ± 3.87 | 0.73 ± 0.32 | 1.45 ± 0.37 | 0.45 ± 0.28 | 28.82 ± 14.53 | 128.81 ± 51.78 |
| | EM | 5.35 ± 1.86 | 0.45 ± 0.15 | 1.23 ± 0.32 | 0.20 ± 0.11 | 40.80 ± 11.20 | 169.89 ± 42.44 |
| | PM | 12.12 ± 1.80 | 1.01 ± 0.15 | 1.68 ± 0.26 | 0.70 ± 0.12 | 16.83 ± 2.39 | 87.72 ± 12.22 |
| Wang | Total | 33.40 ± 8.11 | 2.78 ± 0.68 | 3.08 ± 0.67 | 2.39 ± 0.67 | 6.35 ± 1.54 | 36.54 ± 8.48 |
| | EM | 26.56 ± 3.64 | 2.21 ± 0.30 | 2.52 ± 0.30 | 1.83 ± 0.30 | 7.65 ± 0.96 | 43.76 ± 5.26 |
| | PM | 40.24 ± 4.82 | 3.35 ± 0.40 | 3.64 ± 0.40 | 2.96 ± 0.40 | 5.04 ± 0.58 | 29.33 ± 3.25 |
| Dolton | Total | 35.44 ± 23.6 | 2.95 ± 1.96 | 3.74 ± 2.01 | 2.36 ± 1.83 | 9.06 ± 6.78 | 46.00 ± 29.42 |
| | EM | 24.93 ± 18.11 | 2.07 ± 1.5 | 2.88 ± 1.67 | 1.53 ± 1.34 | 12.24 ± 7.86 | 60.06 ± 32.89 |
| | PM | 45.96 ± 24.13 | 3.83 ± 2.01 | 4.60 ± 1.99 | 3.18 ± 1.91 | 5.88 ± 3.32 | 31.94 ± 16.68 |
| Yun | Total | 102.05 ± 63.53 | 8.50 ± 5.29 | 9.63 ± 5.30 | 7.30 ± 5.03 | 3.16 ± 2.32 | 17.42 ± 12.09 |
| | EM | 48.79 ± 29.69 | 4.07 ± 2.47 | 2.55 ± 2.31 | 3.05 ± 2.31 | 5.14 ± 2.22 | 27.50 ± 15.05 |
| | IM | 89.34 ± 39.14 | 7.44 ± 3.26 | 8.59 ± 3.46 | 6.24 ± 2.97 | 2.89 ± 1.78 | 16.43 ± 10.02 |
| | PM | 179.03 ± 34.33 | 14.92 ± 2.86 | 15.92 ± 3.02 | 13.50 ± 2.59 | 1.16 ± 0.24 | 6.82 ± 1.41 |
| Ling | Total | 51.30 ± 28.27 | 4.28 ± 2.36 | 4.75 ± 2.40 | 3.66 ± 2.27 | 4.97 ± 2.38 | 28.07 ± 12.36 |
| | EM | 41.59 ± 17.30 | 3.47 ± 1.44 | 3.89 ± 1.44 | 2.92 ± 1.43 | 5.34 ± 1.72 | 31.02 ± 9.48 |
| | IM | 49.44 ± 25.70 | 4.12 ± 2.14 | 4.51 ± 2.15 | 3.60 ± 2.11 | 4.84 ± 1.84 | 27.85 ± 10.41 |
| | PM | 62.05 ± 35.90 | 5.17 ± 2.99 | 5.76 ± 3.03 | 4.41 ± 2.88 | 4.64 ± 3.29 | 25.54 ± 16.11 |

Values are presented as mean ± standard deviations from standard dosing regimen (400 mg twice followed by 200 mg twice daily). AUCss, area under the concentration–time curve at steady state; Cavg, average concentration (AUCss/τ); Cmax, maximum concentration; Ctrough, minimum concentration; CL/F, apparent clearance; Vss/F, apparent steady-state volume of distribution.
